# Editorial: Advancements in bioactive nanomaterials for disease management

**DOI:** 10.3389/fphar.2026.1951155

**Published:** 2026-08-06

**Authors:** Peng Liu, Junyong Wu, Jessica C. Hsu

**Affiliations:** 1 Department of Nuclear Medicine, Xiangya Hospital, Central South University, Changsha, Hunan, China; 2 Key Laboratory of Biological Nanotechnology, NHC., Changsha, Hunan, China; 3 FuRong Laboratory, Changsha, Hunan, China; 4 Department of Pharmacy, The Second Xiangya Hospital, Central South University, Changsha, China; 5 School of Health Sciences, College of Health and Human Sciences, Purdue University, West Lafayette, IN, United States

**Keywords:** bioactive nanomaterials, disease management, nanomedicine, precision medicine, targeted drug delivery, theranostics

## Introduction

The integration of nanotechnology and biomedical science is redefining disease management, with bioactive nanomaterials emerging as a particularly promising class of therapeutic platforms. Unlike conventional drug carriers, these engineered constructs interact directly with biological systems: they can modulate cellular signaling, neutralize pathological mediators, and adapt to disease-specific microenvironments. Their high surface-to-volume ratios, tunable surface chemistry, stimuli-responsive properties, and multifunctional capabilities make them particularly well suited to addressing current challenges in precision therapy. The material designs have progressed from simple carriers to sophisticated formulations that incorporate active targeting ligands, environment-responsive moieties, and both diagnostic and therapeutic functions within a single system. Against this rapidly evolving backdrop, the Research Topic, ”*Advancements in Bioactive Nanomaterials for Disease Management*,” brings together fourteen contributions, encompassing original research articles and authoritative reviews that reflect the breadth and momentum of the field. Collectively, these reports examine emerging nanomaterial-based strategies for cancer, inflammatory and immune-mediated disorders, organ-specific diseases, and antimicrobial applications, while highlighting the design principles, therapeutic mechanisms, and translational considerations likely to shape the next-generation of bioactive nanomedicine.

## Cancer nanotherapeutics: multimodal and theranostic strategies

Cancer therapy has long benefited from advances in nanotechnology, and the studies featured in this Research Topic reflect a clear shift toward multimodal theranostic platforms that seamlessly integrate diagnostic and therapeutic functions within a single nanosystem. Han et al. provided a comprehensive review of functional nanomaterials for tumor photothermal therapy, including polydopamine, semiconductor nanoparticles, gold nanomaterials, palladium nanosheets, and carbon-based agents. Their analysis underscores the mechanistic interplay between photothermal conversion and immunogenic cell death while critically assessing emerging combination strategies that pair photothermal therapy with chemotherapy, immunotherapy, or radiotherapy to enhance antitumor efficacy (Han et al.).

Moving beyond monotherapeutic approaches, Luo et al. developed uPAR-targeted, indocyanine green-loaded ferrovirus iron oxide nanoparticles (uPAR-ICG-FVIOs) for dual-modal fluorescence and magnetic resonance imaging to guide magnetic hyperthermia therapy in pancreatic cancer, a malignancy characterized by marked therapeutic resistance and a dense desmoplastic stroma. Their preclinical findings demonstrate the feasibility of combining diagnostic imaging and localized treatment within a single bioactive nanoplatform, thereby exemplifying the theranostic paradigm (Luo et al.). Shi et al. similarly engineered a glutathione-responsive mPEG-b-P(HPMA)-SGI1776 conjugate that exploits redox differences between tumor and healthy tissues to trigger intracellular drug release, resulting in improved pharmacokinetic performance and enhanced therapeutic efficacy in osteosarcoma models (Shi et al.).

Complementing these therapeutic advances, Li et al. developed superparamagnetic nanocarriers coupled with a high-sensitivity mixing-frequency, narrow-band magnetic particle imaging system. This quantitative imaging technique enables stable, real-time tracking of nanocarrier biodistribution, addressing a critical requirement for the clinical translation of nanomedicine (Li et al.).

## Inflammation, immune modulation, and tissue repair

Several contributions examine the use of bioactive nanomaterials to regulate immune responses and treat inflammatory diseases. Li et al. demonstrated that hydroxyethyl starch-curcumin nanoparticles effectively ameliorated DSS-induced ulcerative colitis through a coordinated, multitarget mechanism. The nanoparticles suppressed TLR4/NF-κB-mediated inflammation, activated the Nrf2/HO-1 antioxidant pathway, and restored intestinal barrier integrity by increasing the expression of the tight junction proteins claudin-1, occludin, and ZO-1. They also favorably altered the gut microbiota, including by enriching beneficial *Lactobacillus* species. These findings highlight the therapeutic potential of nanomedicines derived from natural compounds, particularly for diseases involving interconnected inflammatory, oxidative, barrier, and microbial disturbances (Li et al.). Moreover, Wang et al. developed green-synthesized silver nanoparticles using *Colocasia esculenta* and incorporated them into chitosan-based transdermal patches. In a carrageenan-induced paw edema model, the formulation showed greater anti-inflammatory activity than diclofenac sodium, suggesting that it may offer a sustainable and cost-effective approach to the topical treatment of inflammation (Wang et al.).

The therapeutic potential of bioactive nanomaterials also extends to chronic degenerative and age-related inflammatory conditions. Zhu et al. reviewed recent advances in quercetin-based nanomedicines for osteoarthritis and rheumatoid arthritis. They emphasized how polymeric nanoparticles, lipid-based carriers, and inorganic nanoplatforms can improve the otherwise limited bioavailability of quercetin while facilitating its targeted delivery to inflamed joint tissues (Zhu et al.). Lastly, Choi et al. identified green tea-derived exosome-like nanoparticles as potent modulators of oxidative stress-induced skin senescence. By regulating p38 MAPK signaling, these plant-derived nanovesicles attenuated cellular changes associated with ultraviolet-induced skin aging. These findings reveal a potential mechanism through which naturally derived nanomaterials may support skin repair and point to new opportunities for nanomaterial-based dermatological interventions (Choi et al.).

## Organ-targeted nanomedicine: precision delivery to specific tissues

Precision delivery enabled by bioactive nanomaterials is particularly valuable in organ-specific therapies, where distinct anatomical and physiological barriers can limit drug delivery. Yuan et al. developed polysorbate 80-functionalized pullulan nanoparticles for the brain-targeted delivery of paclitaxel. This formulation enhanced penetration of the blood-brain barrier, overcoming a major and longstanding challenge in the treatment of neurological diseases (Yuan et al.). Moreover, Wu et al. demonstrated the therapeutic benefits of linagliptin-loaded chitosan nanoparticles for renal fibrosis, using the mucoadhesive properties of chitosan to promote sustained drug retention in renal tissue (Wu et al.).

In another example, Wang et al. developed biomimetic mesoporous silica nanoparticles loaded with astragaloside IV for the treatment of dilated cardiomyopathy. Their study illustrates how engineered silica-based carriers can protect labile bioactive compounds while facilitating their controlled release in cardiac tissue (Wang et al.). Lastly, Zhao et al. provided a comprehensive review of nanomaterial-enabled systems for delivering plant-derived bioactive metabolites in diabetic retinopathy. Their analysis systematically addresses the challenge of crossing the blood-retinal barrier and highlights formulation strategies designed to improve ocular bioavailability and support clinical translation (Zhao et al.).

## Antimicrobial nanomaterials and multi-disease platforms

The growing global threat of antimicrobial resistance underscores the urgent need for innovative therapeutic strategies capable of circumventing conventional resistance mechanisms. Saha et al. provided a comprehensive overview of antifungal nanomaterials, explaining how metal-based nanoparticles, metal oxides, and carbon-based nanomaterials exert antifungal effects through mechanisms such as membrane disruption, oxidative stress induction, and biofilm inhibition (Saha et al.). Extending beyond antimicrobial applications, Li et al. reviewed nanotechnology-enabled approaches for delivering luteolin, a bioactive compound whose therapeutic potential is limited by poor solubility and rapid metabolism. Their analysis demonstrates how nanocomposite formulations can improve luteolin’s bioavailability and therapeutic efficacy across a range of conditions, including cancer, diabetes, cardiovascular disease, and neurological disorders (Li et al.).

## Concluding remarks and future perspectives

Collectively, the articles assembled in this Research Topic underscore several emerging paradigms in the design of bioactive nanomaterials. These include the transition from single-function carriers to multimodal theranostic platforms that integrate diagnosis, therapy, and real-time monitoring within a single nanoscale construct. They also demonstrate a shift toward nanoplatforms whose constituent materials contribute directly to therapeutic activity—for example, through the anti-inflammatory activity of curcumin, the antioxidant capacity of green tea polyphenols, and the immunomodulatory effects of chitosan—rather than functioning solely as inert carriers for encapsulated drugs. Other notable trends include the development of increasingly sophisticated stimuli-responsive systems that leverage tumor-specific features, including acidic pH, elevated glutathione levels, and dysregulated enzymatic activity, as well as the synergistic integration of natural product-derived bioactive compounds with advanced nanocarrier technologies to optimize pharmacokinetic properties and broaden therapeutic windows.

Despite these remarkable advances, substantial barriers to clinical translation remain. Successful clinical adoption will require comprehensive assessments of long-term biocompatibility and biodegradability, scalable and reproducible manufacturing processes, regulatory frameworks tailored to the distinctive physicochemical properties of bioactive nanomaterials, and rigorous evaluation in preclinical models that more accurately recapitulate human disease pathophysiology. The field would also benefit substantially from standardized characterization protocols and reporting guidelines to support cross-study comparisons, enable robust meta-analyses, and clarify the structure-activity relationships that govern nanomaterial behavior in biological systems. The contributions presented in this Research Topic provide a strong scientific foundation for addressing these challenges and help define priorities for future investigation. Continued collaboration among materials scientists, pharmacologists, engineers, and clinicians will be essential to accelerate the translation of bioactive nanomaterials from the laboratory to clinical practice and, ultimately, to realize their full therapeutic potential for patients worldwide.

